# Disposables used cumulatively in routine IVF procedures could display toxicity

**DOI:** 10.1093/humrep/deae028

**Published:** 2024-03-04

**Authors:** Lucie Delaroche, Lucile Besnard, Valérie Ouary, Fabienne Bazin, Guy Cassuto

**Affiliations:** Ramsay Santé, Hôpital Privé de Parly 2, Institut Fertilité Maternité Parly 2, Le Chesnay-Rocquencourt, France; Centre de Biologie Médicale BIOGROUP, Hôpital Privé de Parly 2, Le Chesnay-Rocquencourt, France; Centre de Biologie Médicale BIOGROUP, Hôpital Privé de Parly 2, Le Chesnay-Rocquencourt, France; C2R SAS, Paris, France; HORIANA, Bordeaux, France; Laboratoire Drouot, Paris, France

**Keywords:** human sperm motility assay, IVF, disposable devices, plastics, cumulative, toxicity

## Abstract

**STUDY QUESTION:**

Is there a cumulative toxicity of disposables used in IVF procedures?

**SUMMARY ANSWER:**

A toxicity may be detected when consumables are used cumulatively, while no toxicity is detected when the same consumables are used and tested individually.

**WHAT IS KNOWN ALREADY:**

Many components of items used in IVF laboratories may impair human embryonic development. Consequently, it is necessary to screen all reagents and materials which could be in contact with gametes and embryos. Toxicity tests, such as the mouse embryo assay and the human sperm motility assay (HSMA), are used by manufacturers as quality control tools to demonstrate the safety of their products. This evaluation is currently individually performed for each single consumable. However, during an IVF cycle, several devices are used sequentially, potentially creating a cumulative exposure to chemical contaminants, which could not be detected for individually tested consumables.

**STUDY DESIGN, SIZE, DURATION:**

The objective of this observational study conducted from March 2021 to October 2022 was to evaluate with the HSMA methodology if there was a cumulative toxicity when several disposables are sequentially used. Fourteen categories of consumables currently used in routine IVF procedures were studied, which included devices used for sperm and oocyte collection (cups, condoms, and oocyte aspiration needles), manipulation (flasks, tubes, tips, pipettes, embryo transfer catheters, syringes, and gloves), culture (dishes), and storage (straws).

**PARTICIPANTS/MATERIALS, SETTING, METHODS:**

After obtaining patient consent, the surplus semen assessed as having normal parameters according to the World Health Organization 2010 criteria were used to perform the HSMAs. First, each consumable was tested individually. Then, associations of three, four, and five consumables, previously validated as non-toxic when tested individually, were analyzed. HSMAs were conducted three times to ensure reproducibility, with a defined toxicity threshold of a sperm motility index (SMI) below 0.85 in at least two of three tests.

**MAIN RESULTS AND THE ROLE OF CHANCE:**

Thirty-six references of disposables were first individually tested across 53 lots. Forty-nine (92%) demonstrated compliance. However, four (8%) devices revealed toxicity: one lot of 1 ml syringes, two lots of sperm cups, and one lot of 25 cm^2^ flasks. These four references were excluded from the IVF routine procedures. A total of 48 combinations of consumables were assessed, involving 41 lots from 32 references that were previously individually tested. Among the evaluated combinations, 17 out of 48 (35%) associations exhibited toxicity with a SMI below 0.85 for two of the three tests (n = 8) or all the three tests (n = 9). Notably, three out of 17 (18%) of the three-consumable associations, five out of 16 (31%) of the four-consumable associations, and nine out of 15 (60%) of the five-consumable associations were found not compliant. The toxicity did not originate from a single consumable, because only consumables that were individually pre-validated as non-toxic were included in the combinations, but the toxicity had a cumulative origin. The risk of cumulative toxicity increased with the number of consumables included in the association (Cochran–Mantel–Haenszel statistic, *P* = 0.013).

**LIMITATIONS, REASONS FOR CAUTION:**

The high proportion of non-compliant combinations of disposables can be attributed directly to the extreme rigorous extraction conditions employed during the tests, which could deviate from the conditions encountered in routine clinical use. Also, the methodology employed in the HSMAs (e.g. toxicity extraction duration, sperm concentrations, and protein supplementation of the medium) can influence the sensitivity of the tests.

**WIDER IMPLICATIONS OF THE FINDINGS:**

This study highlights the significance of performing toxicity testing on devices before introducing them into clinical practice. Disposables should be tested individually to detect immediate toxicities and also in combination. Our results advocate rationalizing the number of consumables used in each IVF procedure and re-evaluating the use of glass consumables.

**STUDY FUNDING/COMPETING INTEREST(S):**

This study received fundings from GCS Ramsay Santé pour l’Enseignement et la Recherche (Paris, France) and the Centre de Biologie Médicale BIOGROUP (Le Chesnay-Rocquencourt, France). The authors declare that they have no conflict of interest that could be perceived as prejudicing the impartiality of the reported research.

**TRIAL REGISTRATION NUMBER:**

N/A.

## Introduction

Plastics have become an integral part of modern society, revolutionizing various industries, and fundamentally changing our way of life. Plastics are raw polymers (base resins) typically enhanced with additives, colorants, plasticizers, and fillers (either soluble or insoluble within the polymer matrix). According to the most common definition, plastics are materials made from synthetic polymers. This definition excludes natural plastics that are derived from biological sources, such as natural rubber initially derived from latex, a milky colloid produced by some plants in tropical countries, primarily *Hevea brasiliensis*. However, a broader interpretation of plastics encompasses both natural and synthetic plastics derived from fossil or renewable resources, better reflecting the diversity of plastics in contemporary usage. Plastics possess remarkable properties such as durability, flexibility, and resistance to degradation. These characteristics have made them incredibly versatile and cost-effective, leading to their widespread use in packaging, construction, electronics, healthcare, and numerous other sectors. The production and consumption of plastics have witnessed an exponential growth over the years, with global production surpassing 460 megatons annually in 2019 and this is on track to triple by 2060 (Landrigan *et al.*, 2023).

However, the dark side of plastics begins to emerge as their environmental impact becomes increasingly apparent. Plastics release toxic chemicals, including additives and residual monomers, both during their use and disposal, leading to critical concerns for human health (Landrigan *et al.*, 2023; Meng *et al.*, 2023). Studies in both animals and humans have revealed associations between plastic exposure and adverse reproductive hormone levels (Yang *et al.*, 2011; Sedha *et al.*, 2021; Panagopoulos *et al.*, 2023). Furthermore, evidence suggests that prenatal exposure to plastic-associated chemicals may have long-term consequences for offspring, including developmental abnormalities and increased susceptibility to certain diseases later in life (Seymore *et al.*, 2022).

The efficacy of ART relies heavily on maintaining stringent control over environmental conditions to ensure the optimal development of *in vitro* embryos (Wale and Gardner, 2016; Cairo Consensus Group, 2020). However, the combined toxicity resulting from various environmental pollutants, chemicals, and physical factors presents a significant threat to the viability and health of developing embryos (Scarica *et al.*, 2022). The use of devices during IVF procedures has raised concerns regarding their potential embryotoxic effects. They have the potential to introduce a complex mixture of associated substances into the culture media through leaching processes (McDonald *et al.*, 2008; Olivieri *et al.*, 2012; Gatimel *et al.*, 2016; Togola *et al.*, 2021). Additionally, IVF plastic devices can release volatile organic compounds which accumulate in the incubators and can alter the culture conditions (Cohen *et al.*, 1997; Mortimer *et al.*, 2018; Vardhan and Shukla, 2018; Chu *et al.*, 2020). All these chemicals contaminants can adversely affect gametes and embryos, both in immediate and long-term ways (Fernández-Gonzalez *et al.*, 2004; Alonso-Magdalena *et al.*, 2010; Chianese *et al.*, 2018; Kouakou *et al.*, 2023) even if human embryos have plasticity and self-correction abilities (Ramos-Ibeas *et al.*, 2019; Coticchio *et al.*, 2021).

Consequently, ESHRE and the American Society for Reproductive Medicine (ASRM) suggest that all medical equipment that may come in contact with gametes and embryos should undergo appropriate quality control tests to prevent the release of embryotoxins (ESHRE Guideline Group on Good Practice in IVF Labs *et al.*, 2016; Practice Committees of the American Society for Reproductive Medicine (ASRM) and the Society for Reproductive Biologists and Technologists (SRBT). Electronic address: asrm@asrm.org, 2022). Ensuring the safety of ART requires the development of risk assessment methods that provide functionally relevant assays for quality control testing. The mouse embryo assay (MEA) and the human sperm motility assay (HSMA) are the two primary tests used to evaluate the suitability of reagents and materials for ART, independent of clinical factors (Ackerman *et al.*, 1985; Critchlow *et al.*, 1989; Claassens *et al.*, 2000; Nijs *et al.*, 2009; Long *et al.*, 2018). Currently, these tests are individually performed for each consumable, ensuring that each consumable has a certificate of conformity. However, during an IVF procedure, multiple devices are used consecutively, potentially leading to cumulative exposure to chemical contaminants that may go undetected when assessing for single consumables. To date, the potential cumulative toxic effects of combinations of consumables have not been studied. The aim of this study was to evaluate the cumulative toxicity resulting from the use of consumables used during an IVF procedure.

## Materials and methods

### Study design

This study was conducted from March 2021 to October 2022 at the Fertility and Maternity Institute, Private Hospital of Parly 2, Le Chesnay-Rocquencourt, France, and was approved by the French South-West and Overseas Personal Protection Committe 4 on 11 December 2020 (Protocol Number ID-RCB: 2020-A01972-37).

### Semen samples

Informed consent was obtained from patients aged between 18 and 65 years who underwent diagnostic sperm tests, granting permission to use any surplus semen samples. Semen samples analyzed in compliance with the Björndahl guidelines and assessed as having normal parameters according to the World Health Organization (WHO) 2010 were included in the study (World Health Organization, 2010; Björndahl *et al.*, 2016). These criteria encompassed a minimum concentration of 15 million/ml or a total number of spermatozoa in the ejaculate of at least 39 million, a progressive motility of at least 32% or a total motility of at least 40%. Within 1 h of collection, the samples underwent processing using a discontinuous density gradient technique with two layers of 40/80% Isolate^®^ (Irvine Scientific, Irvine, CA, USA) for 20 min at 350 g, followed by washing in MHM-C^®^ (Irvine Scientific, Irvine, CA, USA) for 10 min at 200 g. After selection, samples with a total number of progressive motile spermatozoa recovered from ejaculate exceeding 10 million and a progressive motility of at least 70% were used to perform the HSMAs. The final sperm preparation had an adjusted concentration of 4 million/ml motile progressive spermatozoa. The same sperm preparation could be used several times to test individual consumables and/or combinations of consumables.

### Disposables

The study included 14 categories of disposables used in routine IVF procedures, provided by major companies in the field. They were devices used for sperm and oocyte collection (cups, condoms, and oocyte aspiration needles), manipulation (flasks, tubes, tips, pipettes, embryo transfer catheters, syringes, and gloves), culture (dishes), and storage (straws). Some of the included consumables lacked a certificate of conformity or CE (conformity with European health, safety, and environmental protection standards) marking from manufacturers. Mainly used in andrology procedures, these consumables could, however, have been used as a temporary alternative in IVF procedures in the event of stock shortages, subject to prior validation by HSMAs, particularly in the context of the supply difficulties caused by the coronavirus disease 2019 crisis.

Table 1 provides a comprehensive list of the consumable categories, anonymized references (a reference is a model of a consumable that has a specific trade name known under a specific code), and lot numbers, published HSMA/MEA certificates, and CE marking.

**Table 1.** Details of 53 disposable items individually assessed for toxicity using the human sperm motility assay method.

Consumable category	Consumable name	Reference	Lot number	Composition	Use	MEA certificate	HSMA certificate	CE marking	ID graphic
Condom	Spermicid free condom	A	A1	PU	Andrology and IVF	No	No	No	1
Cryotube	Cryotube vials	A	A1	PP (included cap)	Andrology	No	No	No	2
Catheter	Soft Catheter	A	A1	PE	IVF	Yes	No	Yes	3
		B	B1	PP	IVF	Yes	No	Yes	4
Dish	100 mm dish	A	A1	PS Non-treated surface	IVF	Yes	Yes	Yes	5
			A2						6
	35 mm dish	A	A1	PS Non-treated surface	IVF	Yes	Yes	Yes	7
	4 well dish	A	A1	PS Non-treated surface	IVF	Yes	Yes	Yes	8
			A2						9
			A3						10
	60 mm dish	A	A1	PS Non-treated surface	IVF	Yes	Yes	Yes	11
	ICSI dish	A	A1	PS Non-treated surface	IVF	Yes	Yes	Yes	12
Flask	25 cm^2^ flask	A	A1	PS Treated surface (body) Phenol-formaldehyde (cap)	Andrology and IVF	No	No	No	14
			A2						15
		B	B1	PS Treated surface (body) PE (cap)	Andrology and IVF	No	No	No	13
Follicle aspiration needle	Follicule aspiration set	A	A1	Stainless steel (all metal parts) Polyolefin based TPE (tubing) Silicone rubber (plug) MABS polymer (luer connectors) Epoxy resin (glue)	IVF	Yes	No	Yes	16
		B	B1	Steel (needle) PVC (connection tube) ABS polymer TheroPlastic polymer Crosslinked polyolefin Silicone	IVF	Yes	No	Yes	17
		C	C1	Stainless steel (needle) PU (puncture tubing and vacuum tubing)	IVF	Yes	No	Yes	18
Glove	Examination gloves	A	A1	Nitrile	IVF	No	No	Yes	19
	Surgical gloves	B	B1	Natural rubber latex with nitrile polymer	IVF	No	No	Yes	20
Pasteurette	Pasteur pipette 3 ml	A	A1	PE	IVF	Yes	Yes	Yes	22
			A2						23
		B	B1	PE	IVF	Yes	Yes	Yes	21
		C	C1	PE	Andrology	No	No	No	24
Serological pipette	Serological pipette 10 ml	A	A1	PS	Andrology	No	No	No	25
	Serological pipette 5 ml	A	A1	PS	IVF	Yes	Yes	Yes	26
Sperm cup	Sperm collection cup	A	A1	PP (cap and cup) Non-treated surface	Andrology and IVF	Yes	Yes	Yes	27
			A2						28
			A3						29
		B	B1	PS (body) PE (cap)	Andrology and IVF	Yes	Yes	Yes	30
			B2						31
			B3						32
Sperm straw	Sperm straw	A	A1	Ionomer resin. Braided cap (composition not disclosed by the manufacturer)	IVF	Yes	No	Yes	33
			A2						34
Syringe	Syringe 1 ml	A	A1	PP (body and piston) PI (seal)	IVF	Yes	Yes	Yes	37
		B	B1	PP (body and piston) Silicone (seal)	IVF	Yes	No	Yes	35
		C	C1	PP (body) PE (piston) No seal	IVF	No	No	Yes	36
Tips	Cones 1000 µl	A	A1	PP	Andrology and IVF	No	No	No	39
		B	B1	PP	Andrology	No	No	No	38
		C	C1	PP	Andrology and IVF	No	No	Yes	40
	Cones 0.5–200 µl	A	A1	PP	Andrology and IVF	No	No	No	41
Tube	Andrology tube 5 ml	A	A1	PS Non-treated surface	IVF	Yes	Yes	Yes	44
		B	B1	PS Non-treated surface	Andrology and IVF	No	No	No	42
			B2						43
	Centrifuge tube 15 ml	A	A1	PP Non-treated surface	Andrology and IVF	Yes	Yes	Yes	45
			A2						46
		B	B1	PS Non-treated surface	Andrology and IVF	Yes	Yes	Yes	50
	OPU tube	A	A1	PS Non-treated surface	IVF	Yes	Yes	Yes	47
			A2						48
			A3						49
		B	B1	PP	IVF	No	No	No	51
Vitrification straw	Vitrification straw	A	A1	Ionomer resin	IVF	Yes	No	Yes	52
		B	B1	Ionomer resin	IVF	Yes	No	Yes	53

CE: conformity with European health, safety, and environmental protection standards; OPU: Ooocyte pickup; HSMA: human sperm motility assay; MEA: mouse embryo assay; PE: polyethylene; PI: polyisoprene; PP: polypropylene; PS: polystyrene; PU: polyurethane.

Treated surface: surface coating involves altering the surface characteristics of a product to enhance its ability to either adhere to or repel liquids. During the treatment process, the surface undergoes a high-voltage electric shock, which induces a change in the surface energy of the material. This modification imparts adhesive properties, allowing a drop of water placed on the treated surface to spread out and form a flatter shape. In contrast, a drop of water on an untreated surface may retain a more spherical shape but can easily detach when the dish is moved.

### HSMA

All experiments were conducted at a room temperature of 20–24°C (Meintjes, 2017). Initially, single consumables were screened individually for toxicity. Following this, combinations of three, four, or five consumables were tested. A consumable could be included in combinations only if it demonstrated no toxicity during individual testing. Each individual item or combination of disposables was tested three times to ensure the reproducibility of the results. As the volume of one semen preparation was not sufficient to test three items or three associations of items at the same time, HSMAs were conducted using one distinct semen sample for each item or association of items separately. Also, the number of patients who had given their consent or the number of semen samples that met the inclusion criteria was insufficient to conduct tests on all three items or associations on the same day. Consequently, the experiments were performed on different days.

#### Extraction of toxicity

All materials and supplies used to perform the HSMAs were common to the test and the control groups or were known to be non-toxic.

The extraction protocols for assessing the toxicity of the consumables were developed using established methodologies as described in previous studies (Nijs *et al.*, 2009; Meintjes, 2017) (Table 2). For sperm cups and gloves, HSMAs were also performed with alternative extraction conditions that are indicated in italics.

**Table 2.** Summary of human sperm motility assay extraction protocols, by consumable category.

Consumable category		Exposure method	Exposure duration	Volume of sperm suspension
Catheter		Filled with sperm suspension	30 min	0.4 ml
Condom				2 ml
Cryotube			24 h including 30 min in the cap	1 ml
Cup		Filled with sperm suspension	24 h including 30 min in the cap *(30 min excluding the cap)*	1 ml
Dish	35, 60, 100 mm and ICSI	Surface contact with sperm suspension	30 min in the bottom then 30 min in the cap	2 ml
	4 wells	2 wells filled with sperm suspension	24 h	2 × 0.5 ml
Flask		Filled with sperm suspension	24 h including 30 min in the cap	1 ml
Follicle aspiration needle		10 × flushing with sperm suspension then filled with sperm suspension	30 min	
Gloves		0.5 × 0.5 cm sample in sperm suspension	5 min *(1, 10, 30 min)*	
Pasteurette		10 × flushing with sperm suspension then filled with sperm suspension	30 min	
Serological pipette		Filled with sperm suspension		1.5 ml
Sperm straw				0.4 ml
Syringe				1 ml
Tip	1000 µl	10 × flushing with sperm suspension then filled with sperm suspension		
	0.5–200 µl			0.2 ml
Tube	5 ml	Filled with sperm suspension	24 h including 30 min in the cap	1 ml
	14 or 15 ml		30 min in the bottom then 30 min in the cap	
Vitrification straw		Surface contact with sperm suspension	30 min	

Alternative extraction conditions are indicated in italics.

**Figure 1.**
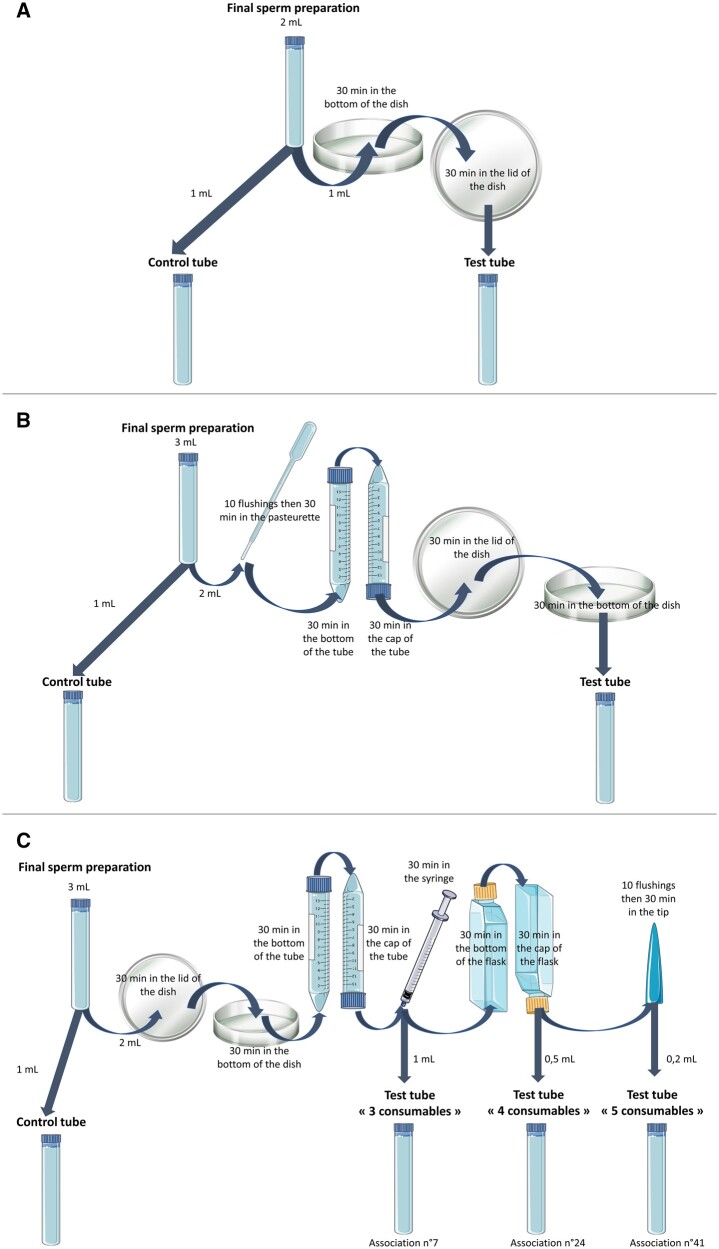
**Toxicity extraction methodologies of the human sperm motility assays.** (**A**) Individually tested consumable. Example of a dish. (**B**) Association of three consumables. Example of an association involving a pasteurette, a tube, and a dish. (**C**) Association of consumables. Interconnexion of three, four, and five consumables. Example of an associations involving a dish, a tube, a syringe, a flask, and a tip.

To test an individual consumable, the test item was either loaded with the sperm preparation or placed into the sperm suspension at room temperature for a specified duration (Fig. 1A). The exposure duration was determined to ensure the consumables were tested under rigorous/stringent extraction conditions, with a minimum exposure time of 30 min, except for gloves. For tubes and dishes, the lids and caps were also tested for a minimum of 30 min. The volume of the sperm preparation used to test consumables depended on the category of consumables; it had to be sufficient to avoid evaporation during incubation, but just enough to avoid diluting toxicity. After the extraction period, the sperm preparation was returned to the test tube of the same reference (same trade name, lot number, and expiry date) as the control tube. The control tube contained the other half of the sperm preparation which remained unchanged. Both tubes were incubated at room temperature for 24 h.

To evaluate combinations of consumables, the sperm preparation was successively brought into contact with each of the consumables included. The duration and extraction method of each consumable included in the combinations were identical to those used for individual testing, with the exception that the 24-h exposure period was reduced to 30 min owing to time constraints and to standardize all exposure durations. Likewise, the volume of the sperm preparation used to test a combination of consumables was adjusted (1 ml minimum and 3 ml maximum) to ensure a sufficient volume in the final test tube.

For example, to test an association of three consumables, such as a pasteurette, a tube, and a dish, 3 ml of the sperm preparation was first aspirated for 30 min into the pasteurette. It was then flushed into the tube, which was initially placed upright to test the bottom of the tube for 30 min, then turned upside down to test the cap for 30 min. Subsequently, the sperm preparation was transferred to the lid of a dish and incubated for 30 min before being placed in the bottom of the same dish for an additional 30 min. Following the successive extraction of the three consumables, the sperm preparation was returned to the test tube and incubated for 24 h (Fig. 1B).

The majority of consumables combinations were evaluated independently, with the exception of seven combinations that underwent interconnected testing (Fig. 1C). To elaborate, the same sperm preparation was initially used to assess the toxicity of the combination of three consumables. Subsequently, the sperm preparation was divided, with one portion allocated to the three-consumable test tube, which was then incubated for 24 h. Meanwhile, the remaining portion of the sperm preparation was used to continue the HSMAs experiments with the addition of a fourth consumable. Following this, the sperm preparation was again divided, with one portion allocated to the four-consumable test tube, which was incubated for 24 h. The remaining portion was then used to continue the experiments with the addition of a fifth consumable. At the end of the experiments and after 24 h of incubation, the progressive motilities of the three-, four-, and five-consumable test tubes were assessed and compared to the progressive motility of the unique control.

#### Sperm motility assessment

Following 24 h of incubation at room temperature, the progressive motility of both test and control tubes for each replicate was independently assessed by two operators at 5-min intervals. To ensure the validity of the test, the control tube had to exhibit progressive motility in more than 50% of the spermatozoa.

The sperm motility index (SMI) was calculated by dividing the mean progressive motility of the test sample by the mean progressive motility of the control after the 24-h incubation period. An SMI value exceeding 0.85, observed in at least two out of three tests, confirmed the absence of toxicity (Critchlow *et al.*, 1989).

The reproducibility of motility assessments between operators was verified through monthly internal quality controls and bi-annual external quality controls.

#### Controls

In order to ensure the reliability and robustness of our methodologies, we conducted preliminary tests to assess whether the sequential transfer of the sperm preparation from one consumable to another during the association tests could lead to a mechanical reduction in sperm motility, potentially introducing bias to our results. A series of control experiments was conducted, involving successive mechanical aspirations of sperm preparations. The objective of this successive mechanical pipetting was to replicate the mechanical transfer of the sperm preparation between consumables. The initial sperm preparation was divided into separate tubes, and in each tube, the sperm preparation was successively aspirated either 10, 20, 30, or 40 times. Subsequently, the tubes were incubated at room temperature for 24 h, and the progressive motility of the sperm in each tube was compared to the control sample, which had not undergone any aspiration. In total, six controls were performed with three sperm suspensions, consisting of two 10-pipetting controls, two 20-pipetting controls, one 30-pipetting control, and one 40-pipetting control.

Additionally, the potential impact of the final volume (0.2 or 0.5 ml) on sperm motility was assessed. Similarly, a sperm preparation was divided into two tubes with final volumes of 0.2 and 0.5 ml that were then incubated at room temperature for 24 h.

### Statistical analysis

For each HSMA, the homogeneity of the three sperm samples used was tested as follows: the SD was calculated for each set of three SMIs. Outliers were determined with the Tukey test, which is a nonparametric outlier detection method calculated by creating a ‘fence’ boundary a distance of 1.5 interquartile range beyond the 1st and 3rd quartiles. Any data beyond these fences were considered to be outliers and were excluded from the analyses.

The distribution of SMI SD was compared between compliant HSMAs (tests with a SMI > 0.85) and non-compliant HSMAs using the Wilcoxon test, for single consumables and associations of consumables.

The trend between number of consumables used in the associations and non-compliance with the HSMA test was assessed using the Cochran–Mantel–Haenszel statistic.

## Results

### Semen samples

A total of 826 patients provided their consent to participate in the study. Based on the inclusion criteria, only 239 semen samples were eligible for inclusion and subsequently used for testing individual consumables and/or combinations of consumables. The other 587 samples did not have sufficient sperm parameters or failed the semen collection. The mean sperm parameters are presented in the Supplementary Table S1.

### Controls

No significant differences in progressive motilities were observed among the control groups of four pipetting conditions and of two volume conditions after 24 h of incubation (Supplementary Fig. S1).

### HSMA

The Tukey test considered SMIs to be homogeneous for eight controls, 53 single consumables and 48 associations of consumables. Seven outliers were excluded, two HSMAs on single consumables and five on associations of consumables (Supplementary Fig. S2).

#### HSMA on single consumables

In total, 36 references of consumables were tested across 53 lots.

**Figure 2.**
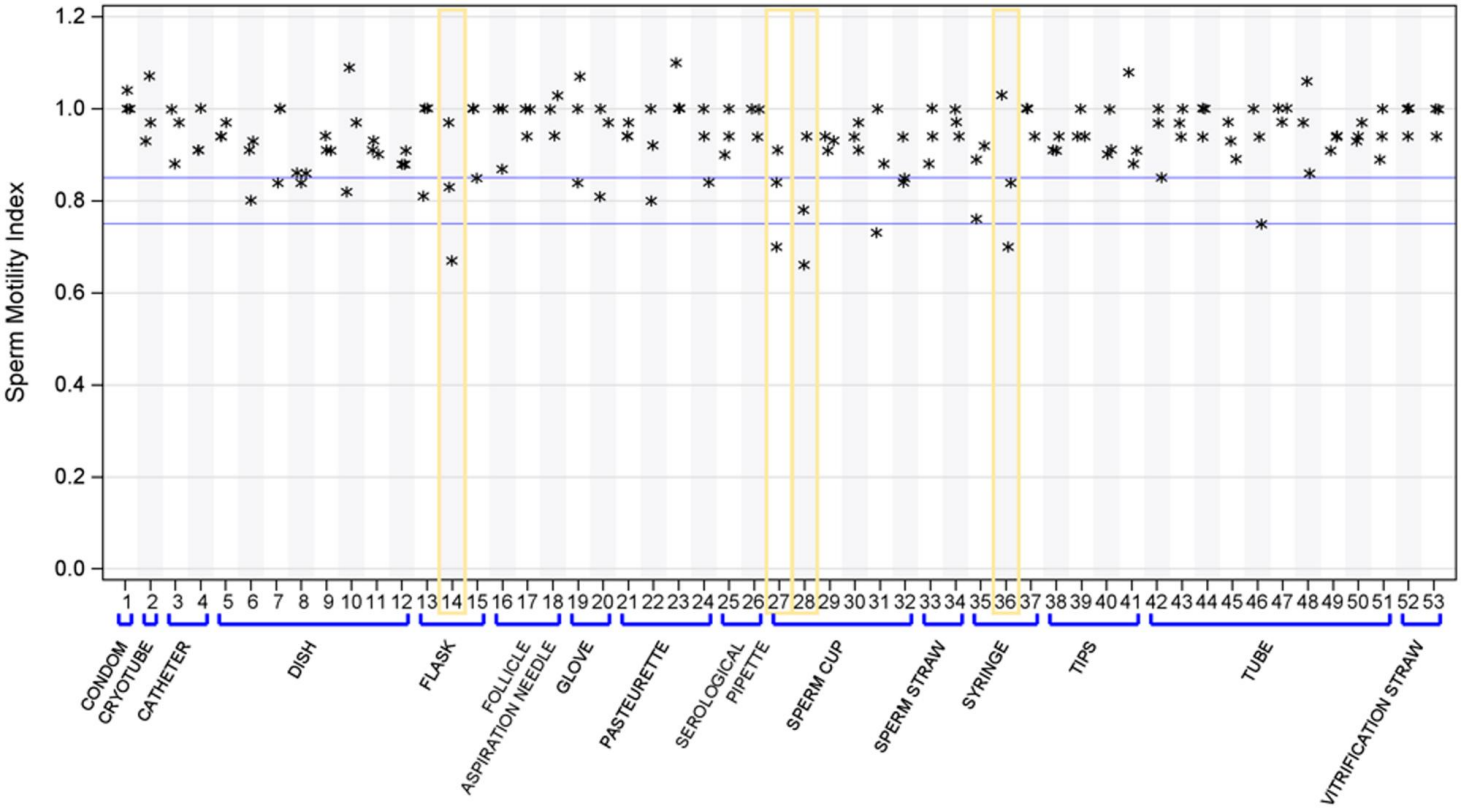
**Sperm motility index results individually tested consumables**.

Forty-nine (92%) items demonstrated compliance with an SMI above 0.85 in at least two out of three tests (Fig. 2). However, four (8%) consumables exhibited toxicity in two out of three tests: the lot A1 of 25 cm^2^ flasks, the lot C1 of 1 ml syringes, and the lots A1 and A2 of sperm collection cups. Consequently, these four references were excluded from our routine andrology and IVF procedures. Of note, two out of these four non-compliant consumables (25cm^2^ flasks and 1 ml syringes) were marketed without MEA or HSMA certificates.

**Figure 3.**
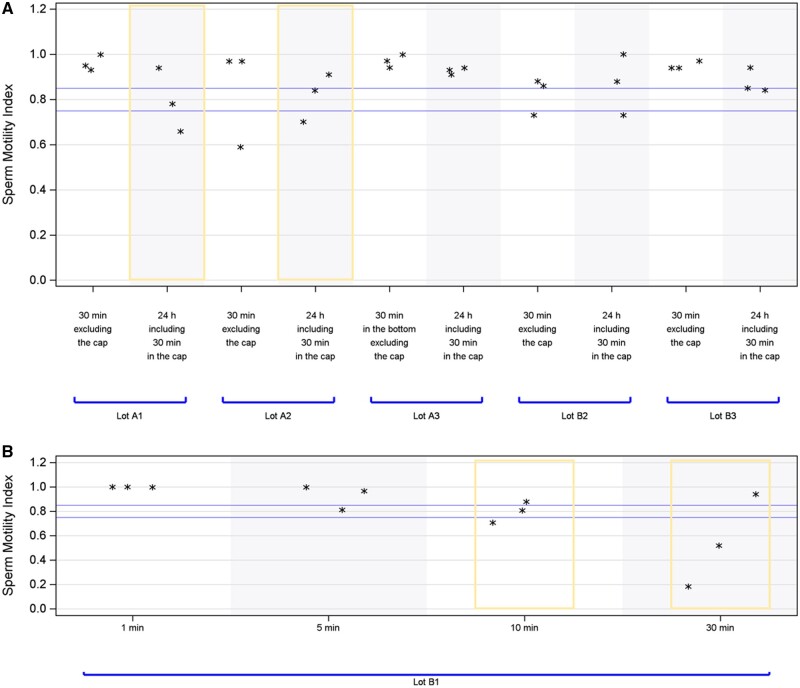
**Sperm motility index results according to the toxicity extraction conditions.** (**A**) Sperm cups. (**B**) Gloves.

For further evaluation, HSMA experiments were repeated on sperm collection cups using alternative extraction conditions. Specifically, the duration of the toxicity extraction for the sperm collection cups was adjusted to 30 min without testing the cap, as opposed to the previous protocol of 24 h of extraction, including 30 min with the cap. This alternative protocol corresponds to the methodology described on the certificate of conformity used by the manufacturer to market the two lots (A1 and A2) which demonstrated toxicity in the initial 24-h extraction conditions. An additional lot (A3) from the same brand and two lots from another brand (B2 and B3) were also implemented and subjected to both the original and modified extraction conditions (Fig. 3A). Remarkably, none of the lots exhibited toxicity when tested under the 30-min extraction condition and without testing the cap.

Moreover, the surgical gloves from lot B1 underwent testing using four different extraction conditions: 1, 5, 10, and 30 min. It was observed that the gloves exhibited toxicity when the extraction duration exceeded 10 min (Fig. 3B).

#### HSMAs on combinations of consumables

A total of 48 combinations of consumables were assessed, consisting of 17 combinations of three consumables, 16 combinations of four consumables, and 15 combinations of five consumables. These combinations involved 41 lots from 32 references that had previously been found compliant when individually tested (Table 3).

**Table 3.** Details of 48 associations of disposable items assessed for cumulative toxicity using the human sperm motility assay method.

Number of association	Number of consumables	Consumable category	Consumable name	Reference	Lot number	MEA certificate	HSMA certificate	CE marking
1	3	Dish	100 mm dish	A	A2	Yes	Yes	Yes
		Pasteurette	Pasteur pipette 3 ml	C	C1	No	No	No
		Tube	OPU tube	A	A2	Yes	Yes	Yes
2	3	Dish	100 mm dish	A	A1	Yes	Yes	Yes
		Pasteurette	Pasteur pipette 3 ml	B	B1	Yes	Yes	Yes
		Tube	Andrology tube 5 ml	B	B1	No	No	No
3	3	Dish	4 well dish	A	A3	Yes	Yes	Yes
			ICSI dish	A	A1	Yes	Yes	Yes
		Pasteurette	Pasteur pipette 3 ml	A	A1	Yes	Yes	Yes
4	3	Dish	100 mm dish	A	A1	Yes	Yes	Yes
			60 mm dish	A	A1	Yes	Yes	Yes
		Tube	Andrology tube 5 ml	B	B1	No	No	No
5	3	Dish	35 mm dish	A	A1	Yes	Yes	Yes
		Pasteurette	Pasteur pipette 3 ml	C	C1	No	No	No
		Tube	Andrology tube 5 ml	A	A1	Yes	Yes	Yes
6	3	Dish	35 mm dish	A	A1	Yes	Yes	Yes
		Tube	Andrology tube 5 ml	A	A1	Yes	Yes	Yes
		Syringe	Syringe 1 ml	B	B1	Yes	No	Yes
7	3	Dish	60 mm dish	A	A1	Yes	Yes	Yes
		Tube	Andrology tube 5 ml	B	B1	No	No	No
			Centrifuge tube 15 ml	A	A1	Yes	Yes	Yes
8	3	Dish	ICSI dish	A	A1	Yes	Yes	Yes
		Pasteurette	Pasteur pipette 3 ml	C	C1	No	No	No
		Tube	Andrology tube 5 ml	A	A1	Yes	Yes	Yes
9	3	Dish	ICSI dish	A	A1	Yes	Yes	Yes
		Pasteurette	Pasteur pipette 3 ml	B	B1	Yes	Yes	Yes
		Tube	Centrifuge tube 15 ml	B	B1	Yes	Yes	Yes
10	3	Dish	100 mm dish	A	A1	Yes	Yes	Yes
		Tube	Andrology tube 5 ml	B	B1	No	No	No
		Sperm cup	Sperm collection cup	B	B3	Yes	Yes	Yes
11	3	Tube	Andrology tube 5 ml	A	A1	Yes	Yes	Yes
			Centrifuge tube 15 ml	A	A1	Yes	Yes	Yes
		Sperm cup	Sperm collection cup	B	B1	Yes	Yes	Yes
12	3	Dish	ICSI dish	A	A1	Yes	Yes	Yes
		Tube	OPU tube	A	A1	Yes	Yes	Yes
		Syringe	Syringe 1 ml	A	A1	Yes	Yes	Yes
13	3	Dish	4 well dish	A	A1	Yes	Yes	Yes
		Tube	Andrology tube 5 ml	A	A1	Yes	Yes	Yes
		Syringe	Syringe 1 ml	B	B1	Yes	No	Yes
14	3	Pasteurette	Pasteur pipette 3 ml	C	C1	No	No	No
		Tube	Andrology tube 5 ml	B	B1	No	No	No
			Centrifuge tube 15 ml	B	B1	Yes	Yes	Yes
15	3	Dish	ICSI dish	A	A1	Yes	Yes	Yes
		Tube	OPU tube	A	A1	Yes	Yes	Yes
		Syringe	Syringe 1 ml	B	B1	Yes	No	Yes
16	3	Tube	OPU tube	B	B1	No	No	No
			Andrology tube 5 ml	A	A1	Yes	Yes	Yes
17	3	Tube	OPU tube	B	B1	No	No	No
			Centrifuge tube 15 ml	B	B1	Yes	Yes	Yes
		Flask	25 cm^2^ flask	B	B1	No	No	No
18	4	Dish	100 mm dish	A	A2	Yes	Yes	Yes
			4 well dish	A	A1	Yes	Yes	Yes
			ICSI dish	A	A1	Yes	Yes	Yes
		Tube	Andrology tube 5 ml	A	A1	Yes	Yes	Yes
19	4	Dish	100 mm dish	A	A1	Yes	Yes	Yes
		Pasteurette	Pasteur pipette 3 ml	B	B1	Yes	Yes	Yes
		Tube	Andrology tube 5 ml	B	B1	No	No	No
		Catheter	Soft catheter	B	B1	Yes	No	Yes
20	4	Dish	4 well dish	A	A1	Yes	Yes	Yes
			ICSI dish	A	A1	Yes	Yes	Yes
			35 mm dish	A	A1	Yes	Yes	Yes
21	4	Dish	100 mm dish	A	A1	Yes	Yes	Yes
			4 well dish	A	A2	Yes	Yes	Yes
			ICSI dish	A	A1	Yes	Yes	Yes
		Tube	Andrology tube 5 ml	A	A1	Yes	Yes	Yes
22	4	Dish	35 mm dish	A	A1	Yes	Yes	Yes
		Pasteurette	Pasteur pipette 3 ml	C	C1	No	No	No
		Tube	Andrology tube 5 ml	A	A1	Yes	Yes	Yes
		Syringe	Syringe 1 ml	B	B1	Yes	No	Yes
23	4	Dish	35 mm dish	A	A1	Yes	Yes	Yes
		Tube	Andrology tube 5 ml	A	A1	Yes	Yes	Yes
		Syringe	Syringe 1 ml	B	B1	Yes	No	Yes
		Flask	25 cm^2^ flask	A	A2	No	No	No
24	4	Pasteurette	Pasteur pipette 3 ml	C	C1	No	No	No
		Tube	Centrifuge tube 15 ml	B	B1	Yes	Yes	Yes
		Sperm cup	Sperm collection cup	B	B2	Yes	Yes	Yes
		Serological pipette	Serological pipette 5 ml	A	A1	Yes	Yes	Yes
25	4	Tube	Andrology tube 5 ml	A	A1	Yes	Yes	Yes
			Centrifuge tube 15 ml	A	A1	Yes	Yes	Yes
		Sperm cup	Sperm collection cup	B	B1	Yes	Yes	Yes
		Sperm straw	Sperm straw	A	A2	Yes	No	Yes
26	4	Dish	100 mm dish	A	A1	Yes	Yes	Yes
			35 mm dish	A	A1	Yes	Yes	Yes
		Pasteurette	Pasteur pipette 3 ml	B	B1	Yes	Yes	Yes
		Tube	OPU tube	A	A3	Yes	Yes	Yes
27	4	Dish	4 well dish	A	A1	Yes	Yes	Yes
		Tube	Andrology tube 5 ml	A	A1	Yes	Yes	Yes
		Syringe	Syringe 1 ml	B	B1	Yes	No	Yes
		Catheter	Soft catheter	A	A1	Yes	No	Yes
28	4	Dish	ICSI dish	A	A1	Yes	Yes	Yes
		Pasteurette	Pasteur pipette 3 ml	C	C1	No	No	No
		Tube	OPU tube	A	A1	Yes	Yes	Yes
		Syringe	Syringe 1 ml	A	A1	Yes	Yes	Yes
29	4	Dish	ICSI dish	A	A1	Yes	Yes	Yes
		Pasteurette	Pasteur pipette 3 ml	C	C1	No	No	No
		Tube	OPU tube	A	A1	Yes	Yes	Yes
		Syringe	Syringe 1 ml	B	B1	Yes	No	Yes
30	4	Dish	100 mm dish	A	A2	Yes	Yes	Yes
			35 mm dish	A	A1	Yes	Yes	Yes
		Pasteurette	Pasteur pipette 3 ml	A	A1	Yes	Yes	Yes
		Tube	OPU tube	A	A2	Yes	Yes	Yes
31	4	Dish	100 mm dish	A	A2	Yes	Yes	Yes
			35 mm dish	A	A1	Yes	Yes	Yes
		Pasteurette	Pasteur pipette 3 ml	A	A1	Yes	Yes	Yes
		Tube	OPU tube	A	A1	Yes	Yes	Yes
32	4	Pasteurette	Pasteur pipette 3 ml	C	C1	No	No	No
		Tube	Centrifuge tube 15 ml	A	A2	Yes	Yes	Yes
		Sperm cup	Sperm collection cup	B	B2	Yes	Yes	Yes
		Serological pipette	Serological pipette 5 ml	A	A1	Yes	Yes	Yes
33	4	Dish	100 mm dish	A	A2	Yes	Yes	Yes
			35 mm dish	A	A1	Yes	Yes	Yes
		Tube	OPU tube	A	A2	Yes	Yes	Yes
			Andrology tube 5 ml	A	A1	Yes	Yes	Yes
34	5	Dish	100 mm dish	A	A2	Yes	Yes	Yes
			4 well dish	A	A1	Yes	Yes	Yes
			ICSI dish	A	A1	Yes	Yes	Yes
		Tube	Andrology tube 5 ml	A	A1	Yes	Yes	Yes
		Vitrification straw	Vitrification straw	B	B1	Yes	No	Yes
35	5	Dish	100 mm dish	A	A1	Yes	Yes	Yes
		Pasteurette	Pasteur pipette 3 ml	B	B1	Yes	Yes	Yes
		Tube	Andrology tube 5 ml	B	B1	No	No	No
		Catheter	Soft catheter	B	B1	Yes	No	Yes
		Tips	Cones 0.5–200 µl	A	A1	No	No	No
36	5	Dish	100 mm dish	A	A1	Yes	Yes	Yes
			60 mm dish	A	A1	Yes	Yes	Yes
		Tube	OPU tube	B	B1	No	No	No
			Andrology tube 5 ml	B	B1	No	No	No
		Syringe	Syringe 1 ml	B	B1	Yes	No	Yes
37	5	Dish	100 mm dish	A	A1	Yes	Yes	Yes
			4 well dish	A	A2	Yes	Yes	Yes
			ICSI dish	A	A1	Yes	Yes	Yes
		Tube	Andrology tube 5 ml	A	A1	Yes	Yes	Yes
		Vitrification straw	Vitrification straw	A	A1	Yes	No	Yes
38	5	Dish	35 mm dish	A	A1	Yes	Yes	Yes
		Tube	Andrology tube 5 ml	A	A1	Yes	Yes	Yes
		Syringe	Syringe 1 ml	B	B1	Yes	No	Yes
		Flask	25 cm^2^ flask	A	A2	No	No	No
		Tips	Cones 0.5–200 µl	A	A1	No	No	No
39	5	Dish	ICSI dish	A	A1	Yes	Yes	Yes
		Pasteurette	Pasteur pipette 3 ml	B	B1	Yes	Yes	Yes
		Tube	Centrifuge tube 15 ml	B	B1	Yes	Yes	Yes
		Syringe	Syringe 1 ml	B	B1	Yes	No	Yes
		Serological pipette	Serological pipette 5 ml	A	A1	Yes	Yes	Yes
40	5	DISH	ICSI dish	A	A1	Yes	Yes	Yes
		Pasteurette	Pasteur pipette 3 ml	C	C1	No	No	No
		Tube	Andrology tube 5 ml	A	A1	Yes	Yes	Yes
			Centrifuge tube 15 ml	A	A1	Yes	Yes	Yes
		Sperm straw	Sperm straw	A	A2	Yes	No	Yes
41	5	Pasteurette	Pasteur pipette 3 ml	C	C1	No	No	No
		Tube	Andrology tube 5 ml	A	A1	Yes	Yes	Yes
			Centrifuge tube 15 ml	A	A2	Yes	Yes	Yes
		Tips	Cones 1000 µl	C	C1	No	No	Yes
		Condom	Spermicid free condom	A	A1	No	No	No
42	5	Dish	100 mm dish	A	A1	Yes	Yes	Yes
		Tube	OPU tube	B	B1	No	No	No
			Andrology tube 5 ml	B	B1	No	No	No
		Sperm cup	Sperm collection cup	B	B3	Yes	Yes	Yes
		Tips	Cones 1000 µl	A	A1	No	No	No
43	5	Dish	100 mm dish	A	A1	Yes	Yes	Yes
			35 mm dish	A	A1	Yes	Yes	Yes
		Pasteurette	Pasteur pipette 3 ml	B	B1	Yes	Yes	Yes
		Tube	OPU tube	A	A3	Yes	Yes	Yes
44	5	Dish	100 mm dish	A	A2	Yes	Yes	Yes
			35 mm dish	A	A1	Yes	Yes	Yes
		Pasteurette	Pasteur pipette 3 ml	A	A1	Yes	Yes	Yes
		Tube	OPU tube	A	A2	Yes	Yes	Yes
45	5	Dish	100 mm dish	A	A2	Yes	Yes	Yes
			35 mm dish	A	A1	Yes	Yes	Yes
		Pasteurette	Pasteur pipette 3 ml	A	A1	Yes	Yes	Yes
		Tube	OPU tube	A	A2	Yes	Yes	Yes
46	5	Pasteurette	Pasteur pipette 3 ml	C	C1	No	No	No
		Tube	Andrology tube 5 ml	B	B1	No	No	No
			Centrifuge tube 15 ml	B	B1	Yes	Yes	Yes
		Syringe	Syringe 1 ml	B	B1	Yes	No	Yes
		Sperm straw	Sperm straw	A	A1	Yes	No	Yes
47	5	Dish	100 mm dish	A	A2	Yes	Yes	Yes
			4 well dish	A	A3	Yes	Yes	Yes
			35 mm dish	A	A1	Yes	Yes	Yes
		Tube	OPU tube	A	A2	Yes	Yes	Yes
			Andrology tube 5 ml	A	A1	Yes	Yes	Yes
48	5	Dish	4 well dish	A	A2	Yes	Yes	Yes
		Tube	OPU tube	B	B1	No	No	No
			Centrifuge tube 15 ml	B	B1	Yes	Yes	Yes
		Flask	25 cm^2^ flask	B	B1	No	No	No
		Serological pipette	Serological pipette 5 ml	A	A1	Yes	Yes	Yes

MEA: mouse embryo assay; HSMA: human sperm motility assay.

**Figure 4.**
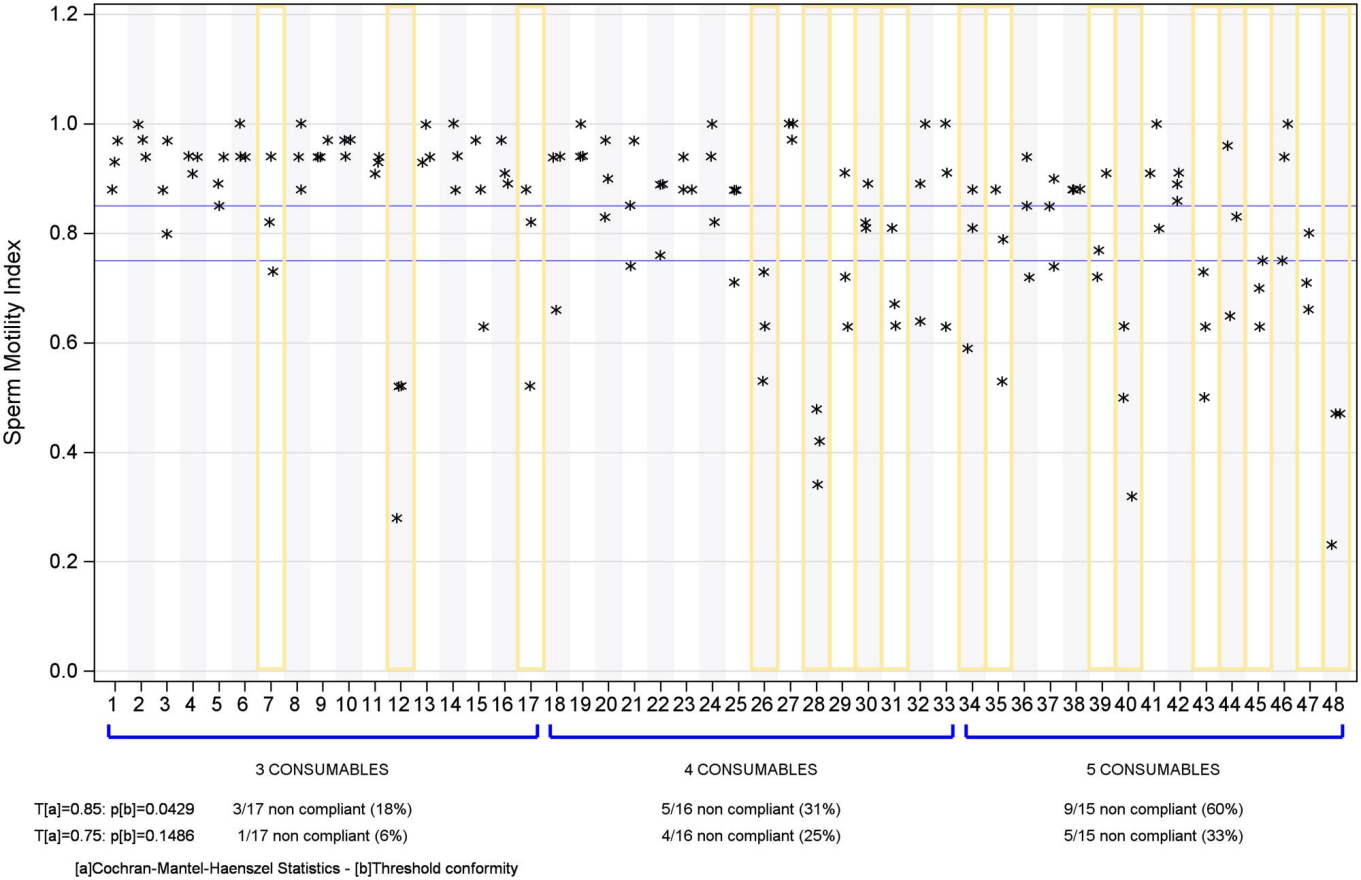
**Sperm motility index results—combinations of disposables**.

Among the evaluated combinations, 17 out of 48 (35%) associations exhibited toxicity with a SMI below 0.85 for two of the three tests (n = 8) or all three tests (n = 9). Notably, three out of 17 (18%) of the three-consumable associations, five out of 16 (31%) of the four-consumable associations, and nine out of 15 (60%) of the five-consumable associations were found not to be compliant (Fig. 4). Thus, the risk of toxicity increased with the number of consumables included in the association (Cochran–Mantel–Haenszel statistic, *P* = 0.013).

Among the 74 items included in the 17 non-compliant associations, dishes were highly frequent (35.4%), especially the lot A1 for 35 mm dishes (9.5% frequency), the lot A1 for ICSI dishes (8.1% frequency), and the lot A2 for 100 mm dishes (8.1% frequency). Additionally, tubes were found accounting for 29.8% of 74 items, while pasteurettes were present at a 14.9% frequency.

For the seven associations that were consecutively tested with the addition of a fourth and/or a fifth consumable, we observed that some combinations which did not show toxicity with three or four consumables became toxic when a fourth or a fifth consumable was added. Also, the associations which were toxic with four consumables remained toxic when a fifth consumable was added. On the other hand, some associations did not become toxic with addition of new consumables (Table 4).

**Table 4.** Results of conformity assessment for seven associations of three, four, and five consumables tested consecutively using the human sperm motility assay method.

**Conformity with three consumables** (Association number)	Conformity when a fourth consumable was added to the same previous association	Conformity when a fifth consumable was added to the same previous association
Compliant	Compliant	Compliant
(7)	(24)	(40)
Compliant	Compliant	Non-compliant
(3)	(20)	(36)
NA	Compliant	Compliant
	(22)	(38)
NA	Compliant	Non-compliant
	(19)	(35)
NA	Compliant	Non-compliant
	(34)	(50)
NA	Non-compliant	Non-compliant
	(27)	(45)
NA	Non-compliant	Non-compliant
	(31)	(46)

NA: non-applicable.

In total, the variability between the three SMIs of a same test was significantly higher for non-compliant tests than for compliant tests, both for consumables alone (Wilcoxon, *P* = 0.002) and for associations of consumables (Wilcoxon, *P* = 0.002). These differences suggest that sperm samples may have different degrees of sensitivity to the toxic effects of consumables.

## Discussion

This study aimed to assess the potential cumulative toxicity arising from combinations of devices used in IVF procedures. Notably, it was found that 17 out of 48 (35%) associations of three, four, or five disposables, tested using the HSMA methodology, exhibited toxicity toward spermatozoa. This toxicity had a cumulative origin: it did not originate from one single consumable because only consumables that were individually pre-validated as non-toxic were included in the combinations, and increased with the number of consumables included in the association. However, the precise source of the toxicity remained unknown. It could potentially stem from various factors, including the materials’ composition, production process, handling and packaging, sterilization, or transport process (Nijs *et al.*, 2009). All these factors could lead to an accumulation of toxic molecules leaching from disposable plasticware into the sperm preparation as it successively came into contact with plastic consumables (McDonald *et al.*, 2008). These molecules could reduce or stop the cell metabolism responsible for motility in human spermatozoa (Nijs *et al.*, 2009). The release of toxic molecules could have a fairly immediate effect on sperm motility as observed in this study, but could be more insidious in case of embryo culture, as it has already been demonstrated, reducing fertilization and blastulation rates or influencing negatively the post-implantation development and the health of newborns (Takai *et al.*, 2001; Susiarjo *et al.*, 2015; Kouakou *et al.*, 2023). The susceptibility to toxicity may differ among biological samples.

Furthermore, we identified that four out of 53 (8%) devices exhibited toxicity when individually tested. This percentage of toxic devices was lower than that reported in a previous study where 13 out of 36 (36%) products were found to be toxic (Nijs *et al.*, 2009). It is important to note that at the time of the Nijs *et al.* (2009) study, the consumables did not possess certificates of conformity. This disparity is reassuring, as it suggests an improvement in the safety of consumables over time. Since the recommendations by the ESHRE and ASRM societies, the majority of IVF devices are now subjected to validation for the absence of toxicity before being introduced to the market (ESHRE Guideline Group on Good Practice in IVF Labs *et al.*, 2016; Practice Committees of the American Society for Reproductive Medicine (ASRM) and the Society for Reproductive Biologists and Technologists (SRBT). Electronic address: asrm@asrm.org, 2022). Suppliers are becoming increasingly aware of the importance of ensuring the safety, reliability, and traceability of their products. For some, this involves controlling both their raw materials and finished products. Meanwhile, a growing number of high-quality IVF laboratories are using the HSMA to assess new disposables and evaluate each lots of accepted disposables. In this study, two out of the four consumables were found to be toxic, specifically the 1 ml syringes and the 25 cm^2^ flasks, which both lacked MEA/HSMA certificates of conformity. This indicates that a consumable that has not undergone prior testing for embryotoxicity is more prone to being identified as toxic. The cap made of phenol and formaldehyde in the 25 cm^2^ flasks could be the source of their toxicity (Thrasher and Kilburn, 2001). Our results further confirmed the previously reported toxicity of gloves (Critchlow *et al.*, 1989; Lierman *et al.*, 2007; Nijs *et al.*, 2009). Even a contact duration of ∼10 min between gloves and the sperm preparation was found to have a significant impact on motility. Caution should be exercised, as previous studies have indicated that rapid contact of gloves with a device could be detrimental owing to the potential detachment of fine particles from the gloves (Critchlow *et al.*, 1989).

Additionally, our study has confirmed that the methodologies employed for toxicity extraction did have a significant impact on the sensitivity of the HSMA. We observed that two lots of sperm collection cups, which exhibited toxicity when tested using a 24-h extraction including 30 min of cap testing, no longer demonstrated toxicity when subjected to a 30-min extraction without cap testing. In a previous study (Delaroche *et al.*, 2020), we identified significant variation among manufacturers in the methodologies used for toxicity testing of IVF disposables, including the MEA and HSMA. Importantly, certain parameters, such as sperm concentration, protein supplementation of the media, and incubation duration, were found to impact the sensitivity of the results (Claassens *et al.*, 2000; Lierman *et al.*, 2007). These methodological differences may contribute to the divergent toxicity outcomes observed for the same consumable. To date, there are no established guidelines for the methodology of conducting HSMAs, unlike MEAs, which have Food and Drug Administration (FDA) recommendations (Mouse Embryo Assay for Assisted Reproduction Technology Devices—Guidance for Industry and Food and Drug Administration Staff, n.d.). These FDA guidelines suggest testing only the parts of the article directly in contact with gametes and embryos during clinical use. Applying these guidelines to HSMAs would imply that the lids or caps of plasticware should not undergo routine testing. However, practical scenarios in IVF laboratories may lead to unavoidable contact between samples and lids or caps. For example, sperm collection containers may occasionally reach the laboratory with the sample touching the cap, and tubes used in oocyte retrievals might be overfilled, causing the cap to contact the follicular fluid. There is also the possibility of a tube accidentally tipping over, with its contents coming into contact with the cap. Furthermore, our rationale for choosing a 30-min contact was based on the FDA guidelines for MEA, which recommend a minimum contact time of 30 min for all consumables not used in embryo culture (Mouse Embryo Assay for Assisted Reproduction Technology Devices—Guidance for Industry and Food and Drug Administration Staff, n.d.). Other guidelines advocate that the exposure duration should be at least double the expected exposure time of sperm to the material (for pipette tips, exposure for a few seconds, and for sample collection receptacles, exposure from 30 to 60 min) (EN ISO 23162:2021, n.d.). The comprehensive testing approach employed for our study was grounded in a rigorous evaluation of toxicity, guided by the principle that a robust bioassay should not only be sensitive but also reliable, reproducible, and consistent in detecting compromised contact materials. Thus, quality controls need to be conducted under conditions that exceed the routine use conditions (Meintjes, 2017). Additionally, the choice of toxicity thresholds can lead to different conclusions regarding compliance. Initially, the toxicity threshold was defined as an SMI above 0.85 after 96 h of incubation (Critchlow *et al.*, 1989). However, the currently accepted toxicity threshold is an SMI above 0.75 after 24 h of incubation (Meintjes, 2017; Delaroche *et al.*, 2020). When the toxicity threshold was lowered to 0.75, no more single consumables and only 10 (21%) associations were found to be toxic in this study.

The majority of IVF consumables included in this study are composed of polymers from the thermoplastics family, specifically polystyrene, polypropylene, and polyethylene. The existing literature suggests a potential decline in fertility associated with environmental exposure to microparticles and nanoparticles derived from these polymers. They may accumulate in mammalian reproductive organs, exerting toxic effects on the reproductive systems of both genders, potentially leading to transgenerational impacts (Afreen *et al.*, 2023; He and Yin, 2024). However, there is a noticeable gap in research concerning the direct toxicity of these polymers on gametes and embryos. In contrast, bisphenols (BPs), particularly bisphenol A (BPA), have been extensively studied. BPs, widely used as plasticizers, are primarily employed in polycarbonate synthesis, which is used in the production of medical devices (Gatimel *et al.*, 2016). Concerns about the impact of BPA exposure on reproductive health outcomes have led to numerous publications based on *in vitro*, animal, and human studies. BPs are known to affect fertility as endocrine disruptors, exhibiting weak estrogenic activity. They also exert epigenetic effects in both male and female reproduction, with implications across multiple generations (Welshons *et al.*, 2006; Susiarjo *et al.*, 2015; Chianese *et al.*, 2018; Ryu *et al.*, 2023). Exposure to BPA during the final stages of meiosis can disrupt the meiotic spindle, cause chromosomal misalignment, increase the incidence of meiotic arrest, and lead to aneuploidy in mouse oocytes (Can *et al.*, 2005). In human studies, a negative dose–response association of BPA exposure with altered oocyte IVM has been observed (Machtinger *et al.*, 2013). Owing to restrictive guidelines, BPA is now being replaced by its structural analogues, bisphenol S (BPS) and bisphenol F (BPF). While their impact on fertility is less well studied, initial reports indicate they also exhibit negative effects on reproductive functions, albeit through distinct intracellular pathways and mechanisms compared to BPA (Rochester and Bolden, 2015; Abouhamzeh *et al.*, 2023). In various animal models, BPS has been shown to disrupt granulosa cell steroidogenesis (Téteau *et al.*, 2020, 2023), impair the *in vitro* early developmental competence of oocytes and embryos (Desmarchais *et al.*, 2020; Žalmanová *et al.*, 2023) and reduce the motility of spermatozoa (Torres-Badia *et al.*, 2023; Téteau *et al.*, 2023). Similarly, BPF has been shown to affect motility, capacitation, and mitochondrial membrane potential in cryopreserved bovine sperm (Nguyen *et al.*, 2022; Davis *et al.*, 2023). In our study, none of the included consumables were made of polycarbonates. Therefore, the toxicity we observed could not be related to the potential leaching of BPs. However, studies have shown that plastic materials, especially strippers, can leach BPA into the surrounding medium (Gatimel *et al.*, 2016). The leaching of BPs, observed at levels below the previously detected upper limit in women’s serum and follicular fluid, did not appear to exert a significant impact on embryo development (Moreau *et al.*, 2021). Moreover, contamination of gametes and embryos by BPs could also originate from culture media (Togola *et al.*, 2021). Togola *et al.* (2021) revealed that the majority of culture media used in IVF procedures contained BPS, with concentrations approaching the nanomolar range. This raises concerns about the potential cocktail effect of culture media and consumables, which has not been adequately investigated to date. Further research is required to fully comprehend the cumulative risks associated with these media and disposables and to develop safer alternatives.

The present investigation has several limitations. First, because of constraints related to the limited availability of semen samples (approximately eight semen diagnoses per day), we could not use a single semen sample for all replicates. Instead, we were limited to using three different semen samples for the three replicates. This limitation may introduce variability in the results owing to natural variations in semen quality. It would have been valuable, when possible, to pool the three semen samples to have a unique final sperm preparation that could be used for the three tests. However, previous research has shown that the influence of semen source on sperm motility assay quality is minimal (Claassens *et al.*, 2000) and to address the issue, we statistically checked the homogeneity between the three semen samples included in each test. The second limitation arises from the possibility that conducting experiments for the same test over multiple days may have influenced the results, even with controlled environmental and technical conditions. Variations in parameters such as temperature or humidity can impact equipment performance and biological reactions, contributing to result variations (Obermeyer and Pope, 2021). Standardized procedures may not eliminate internal variability, as different operators following the same protocols could introduce subtle variations in sample handling (for example accuracy of pipetting) (Crawford *et al.*, 2023). Furthermore, mobility assessments were blindly conducted blind by two operators, and the average of their values was used for analysis. Despite regular internal and external quality checks ensuring a well-controlled process (Siddharth *et al.*, 2023), the variability between operators may explain some random SMI values exceeding 1 in some single consumable tests. Finally, despite the implementation of controls, it was not possible to fully eliminate the potential mechanical influence of transferring the sperm preparation from one consumable to another. It was apparent that a fraction of the sperm preparation was lost with each contact made with the consumables being tested in the associations. To complete our results, it would have been valuable to quantify the cumulative levels of toxins, such as BPs or phthalates, that could be released from associations of devices. Nevertheless, there is currently no exhaustive compilation of molecules resulting from the degradation of plastic and materials. Taking plastic packaging as an example, Meng and colleagues reported that plastic packaging materials were associated with a minimum of 10 259 chemicals, encompassing substances used in manufacturing, such as solvents, non-intentionally added substances, impurities, oligomers, and degradation products (Meng *et al.*, 2023).

In conclusion, our preliminary observations document some cumulative toxicity on spermatozoa from disposables sequentially used in ART procedures. The quite high proportion of non-compliant combinations of devices can be attributed directly to the extreme rigorous extraction conditions employed during the tests, which deviated from the conditions encountered in clinical routines. Further work is warranted to investigate whether the cumulative toxicity observed under strict extraction conditions is still found in the real-life conditions.

Moreover, our study confirms the significance of conducting toxicity testing on consumables before introducing them into routine clinical practice. Integrating internal testing of consumables without a certificate of conformity into ongoing quality control programs is crucial. While individual screening remains essential, consumables should also be evaluated in combination with culture media, using methodologies consistent with real-life clinical practices. Another question raised by our study is the lack of regulation and standardization in HSMA testing protocols. To address the lack of harmonization, we recommend that IVF laboratories conduct testing of their associations of consumables and media under their own established protocols. Finally, this study advocates rationalizing the number of consumables used in each IVF procedure and re-evaluating the use of glass consumables (Kouakou *et al.*, 2023).

Understanding the mechanisms underlying the toxicity of synthetic materials on human health and reproduction is crucial for mitigating their detrimental effects and formulating effective regulatory measures (Eladak *et al.*, 2015). While there has been some progress in acknowledging and restricting the use of certain hazardous plastic and synthetic compounds, further research is required to elucidate the long-term consequences of exposure to synthetic materials and identify alternatives that pose fewer risks.

## Supplementary Material

deae028_Supplementary_Figure_S1

deae028_Supplementary_Figure_S2

deae028_Supplementary_Table_S1

## Data Availability

Relevant data are available in the article and in its supplementary material. The complete database, including complementary data not presented in the article, is recorded on a secured server with limited access only to authorized users. If an access to these complementary data is needed, they can be shared on reasonable request to the corresponding author.
