# Supplementary figures and images for: Disposables used cumulatively in routine IVF procedures could display toxicity
Source: Hum Reprod. 2024 Mar 4;39(5):936–54. doi: 10.1093/humrep/deae028 (PMC11063546; doi:10.1093/humrep/deae028)

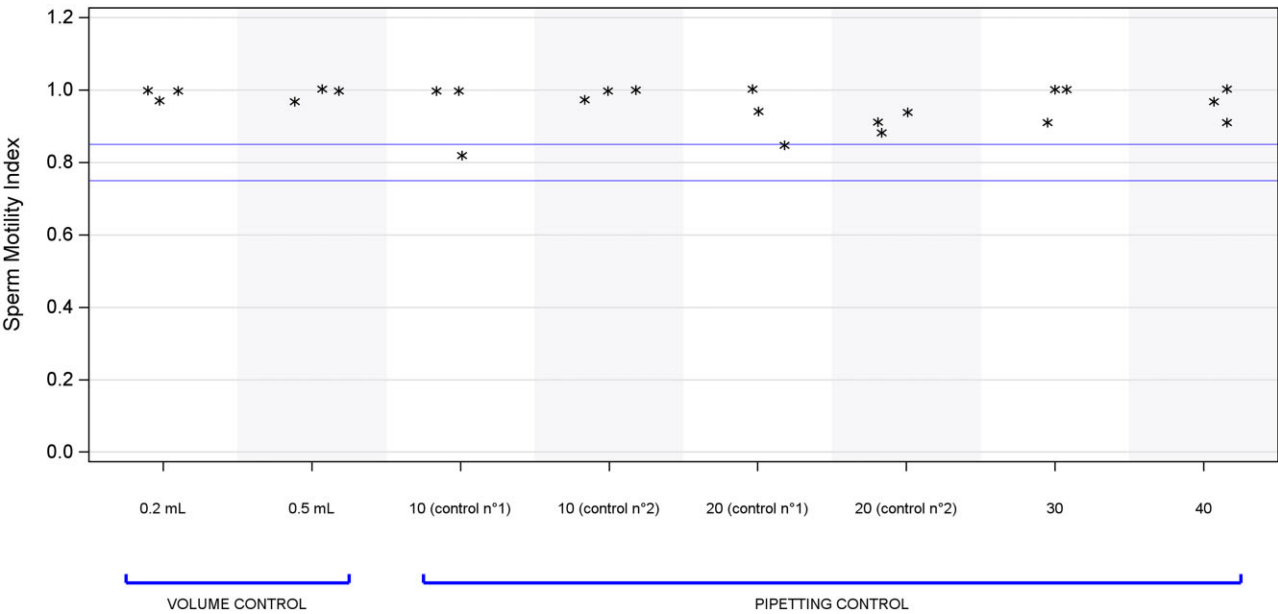

Supplementary Figure S1. Sperm motility index results for control.

Supplement: deae028_Supplementary_Figure_S1 [file deae028_supplementary_figure_s1.pdf]
