# Supplementary material for: Disposables used cumulatively in routine IVF procedures could display toxicity
Source: Hum Reprod. 2024 Mar 4;39(5):936–54. doi: 10.1093/humrep/deae028 (PMC11063546; doi:10.1093/humrep/deae028)
Supplement: deae028_Supplementary_Figure_S2 [file deae028_supplementary_figure_s2.pdf]

OXFORD
human reproduction
Human Reproduction, 2024, 1–1
https://doi.org/10.1093/humrep/deae028
Supplementary Data

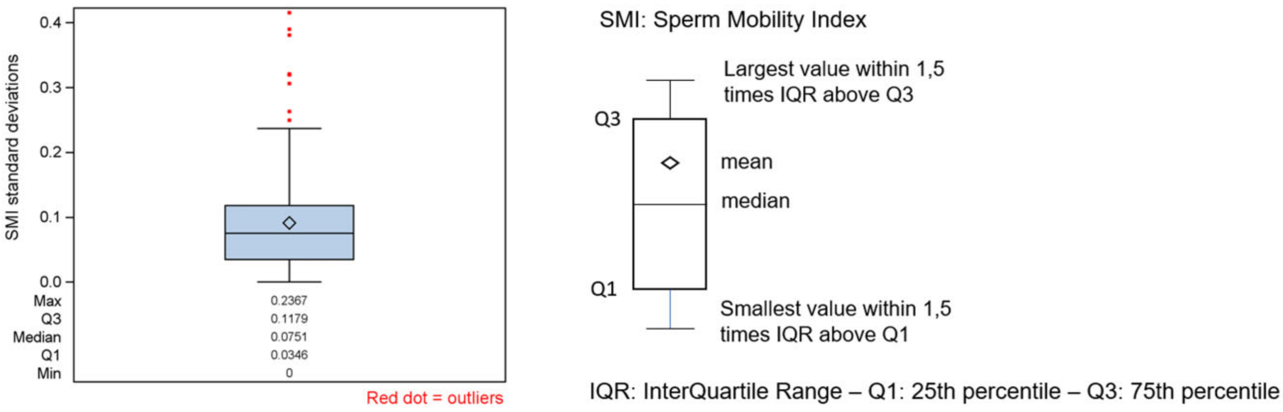

Supplementary Figure S2. Sperm motility index standard deviation distribution.
