# Supplementary material for: Disposables used cumulatively in routine IVF procedures could display toxicity
Source: Hum Reprod. 2024 Mar 4;39(5):936–54. doi: 10.1093/humrep/deae028 (PMC11063546; doi:10.1093/humrep/deae028)
Supplement: deae028_Supplementary_Table_S1 [file deae028_supplementary_table_s1.pdf]

OXFORD human reproduction

*Human Reproduction*, 2024, 1–1
https://doi.org/10.1093/humrep/deae028
Supplementary Data

**Supplementary Table S1.** Semen parameters of the 239 samples included in the study.

| | |
|---|---|
| **Initial sperm parameters** | |
| Concentration ($10^6$/ml) | 116.0 ± 85.8 [27.1–597.0] |
| Total number of sperm per ejaculate ($10^6$) | 406.1 ± 294.3 [56.7–2029.8] |
| Progressive motility (%) | 48.7 ± 6.4 [35.0–65.0] |
| Total motility (%) | 54.0 ± 6.4 [40.0–70.0] |
| **Final sperm parameters after selection** | |
| Progressive motility (%) | 87.4 ± 5.4 [70.0–95.0] |
| Total number of progressive motile sperm recovered from the ejaculate ($10^6$) | 76.3 ± 63.3 [12.8–425.2] |

Mean ± SD [min–max].
